# Nanoparticle-Biological Interactions in a Marine Benthic Foraminifer

**DOI:** 10.1038/s41598-019-56037-2

**Published:** 2019-12-19

**Authors:** Caterina Ciacci, Margot V. Grimmelpont, Ilaria Corsi, Elisa Bergami, Davide Curzi, Debora Burini, Vincent M. P. Bouchet, Patrizia Ambrogini, Pietro Gobbi, Yurika Ujiié, Yoshiyuki Ishitani, Rodolfo Coccioni, Joan M. Bernhard, Fabrizio Frontalini

**Affiliations:** 1 Università degli Studi di Urbino, Department of Biomolecular Science, Urbino, Italy; 2 Université de Lille, CNRS, Université Littoral Cote d’Opale, UMR 8187, LOG, Laboratoire d’Océanologie et de Géosciences, Wimereux, France; 3 Università degli Studi di Urbino, Department of Pure and Applied Science, Urbino, Italy; 4 Università degli Studi di Siena, Department of Physical, Earth and Environmental Sciences, Siena, Italy; 5 Kochi University, Center for Advanced Marine Core Research, Nankoku, Japan; 6 University of Tsukuba, Section Center for Computational Sciences, Tsukuba, Japan; 7 Geology and Geophysics Department, Woods Hole Oceanographic Institution, Woods Hole, MA USA

**Keywords:** Environmental sciences, Environmental impact, Natural hazards

## Abstract

The adverse effects of engineered nanomaterials (ENM) in marine environments have recently attracted great attention although their effects on marine benthic organisms such as foraminifera are still largely overlooked. Here we document the effects of three negatively charged ENM, different in size and composition, titanium dioxide (TiO_2_), polystyrene (PS) and silicon dioxide (SiO_2_), on a microbial eukaryote (the benthic foraminifera *Ammonia parkinsoniana*) using multiple approaches. This research clearly shows the presence, within the foraminiferal cytoplasm, of metallic (Ti) and organic (PS) ENM that promote physiological stress. Specifically, marked increases in the accumulation of neutral lipids and enhanced reactive oxygen species production occurred in ENM-treated specimens regardless of ENM type. This study indicates that ENM represent ecotoxicological risks for this microbial eukaryote and presents a new model for the neglected marine benthos by which to assess natural exposure scenarios.

## Introduction

Engineered nanomaterials (ENM), nanomaterials conceived, designed, and intentionally produced by humans^1^, have been progressively included in commercial and personal-care products for their unique properties (e.g. particle size, surface area, surface reactivity, charge, and/or shape)^2^. ENM are introduced to waterbodies mainly via urban and industrial sewage^3^, and ultimately into coastal marine environments where the highest concentrations are expected to occur in sediments^4^. The marine environment is considered a major sink of anthropogenic contaminants. Therefore, the increasing use and associated emissions of ENM indicate the need for a clearer understanding of their behavior in marine habitats and their effects on biota^5^. The current evaluation of ENM impact on biota relies mainly on *in vitro* studies using animal models; these approaches are important because detection of ENM at trace concentrations in natural samples is, in most cases, not yet possible^6,7^.

Among the metallic ENM, titanium dioxide (TiO_2_) and silica dioxide (SiO_2_) nanoscale particles (NP) are certainly among the most abundant in terms of worldwide production with their major emission pathway to marine coastal waters via wastewater effluents^8^. Estimated TiO_2_ concentrations in surface river waters and sediment are 2.17 µg/L and 43.3 mg/kg, respectively, for the EU (data for 2014)^7^. Coastal marine waters are characterized by high concentrations of ionic and organic matter (i.e., suspended colloidal particles) that interact with TiO_2_ and significantly affect its fate in seawater^9^. Synthetic SiO_2_ has been used as an additive in food and for tires, and its production has greatly increased over the last few decades. Because amorphous SiO_2_ has a low solubility in water, the vast majority of it is expected to settle in soil and sediment^10^.

Similarly, nanopolymers such as polystyrene (PS) have been included in a wide range of applications including biosensors, photonics, nanocomposites and drug delivery (see ref. ^11^ for example). PS debris is commonly found in marine environments as macro-plastic^12,13^, which is likely to degrade to micro- (commonly defined as fragments < 5 mm) and nanoplastics (smaller than 1 μm)^14^. Plastic waste is ubiquitous and is predicted to increase by an order of magnitude in the ocean over the next decade, thus posing a serious threat to marine food webs, including microbes^15–17^. Because plastic is the major component of seafloor litter, as was recently reported in a study on the Adriatic Sea^18^, micro- and nanoplastics occur in marine sediments, where ENM are also accumulated.

ENM environmental fate and behavior are influenced by their unique nanoscale features but also by their interaction with surrounding media^19^. Recent studies reported that hetero agglomeration (i.e., the agglomeration between non-homologous particles) represents a far more significant process than homoagglomeration (e.g., the agglomeration of only NP) for negatively charged NP such as TiO_2_ and SiO_2_ in the presence of relatively high pH and ionic strength as in seawater^20,21^. Nano-scale PS also acquires negative surface charges that could drive a similar heteroagglomeration process in marine coastal waters^22^. Natural organic matter (NOM) has a major effect on agglomeration and confers a new biological identity as, for instance, in the formation of eco-corona^20,23–25^. As a consequence, the high agglomeration of ENM and NP accelerates their sedimentation to the seafloor^21^, leading to potentially critical exposure scenarios for benthic-dwelling species^22,26^. In spite of widespread research to develop applications for ENM, their potential toxicological effects, biological interactions and release have been studied far less frequently^1,10^. Smaller particles are commonly more toxic than larger ones due to their ability to pass biological barriers, bioaccumulate and affect metabolism^27–29^. Most studies of the impacts of ENM use pelagic rather than benthic-dwelling organisms^30^. The uptake of NP by benthic invertebrates serving as food for higher trophic-level organisms may promote transfer of ENM through the food web^31^. Thus, the evaluation of the adverse toxic and physiological effects for benthic marine organisms has become a major concern^32,33^.

Eukaryotes (e.g., metazoans and protists like benthic foraminifera) have evolved the ability to internalize particles. Protists, with their trophic position, short life span, general prevalence of asexual reproduction (*vs*. mutagenic sexual reproduction), and responsiveness, are suitable models to assess the impacts of ENM. Benthic foraminifera are sediment-dwelling, single-celled marine eukaryotes, commonly short-lived and with reproductive cycles that play a central role in global biogeochemical cycles of inorganic and organic compounds (see ref. ^34^ for example). These organisms can ingest nano- and micro-sized particles, for example during seawater endocytosis or in sediments via reticulopodia, which are used for gathering food such as bacteria and algae. Indeed, benthic foraminiferal exposure to pollutants (e.g., trace elements, oil, and drilling muds) induces physiological stress as evidenced by a thickening of the inner organic lining, variation in the number and size of lipid droplets (LD), degeneration of mitochondria, proliferation of degradation vacuoles, reduction in the chamber formation rate, and a decrease in pseudopodial activity (see refs. ^35,36^ for examples).

Despite the recent advances and investigations into the occurrence, distribution, and abundance of ENM, including nanoplastics, in marine environments, there remain relevant knowledge gaps, particularly on their effects on the benthos. No study, to our knowledge, has documented their effect on foraminifera. Therefore, the present study aimed to evaluate the effects of short-term exposure of different types of NP on the benthic foraminiferal species *Ammonia parkinsoniana*, which is a common taxon in some settings. A NP concentration of 1 mg/L was selected based on previous *in vitro* model studies^37^ and because it is well below the recommended limits of 100–1000 mg/L that appear in ENM test guidelines^38^. Using a combination of techniques including transmission electron (TEM), environmental scanning electron (ESEM), coupled with energy dispersive X-ray spectrometry (EDS), and confocal laser scanning (CLSM) microscopies, we show in this *in vivo* experiment the internalization of different NP in the cytoplasm of *A*. *parkinsoniana*, as well as the associated intracellular lipid accumulation, free radical production and the alteration of mitochondria in ENM-treated specimens.

## Results

### Characterizations of nanoparticles

**Figure 1:**
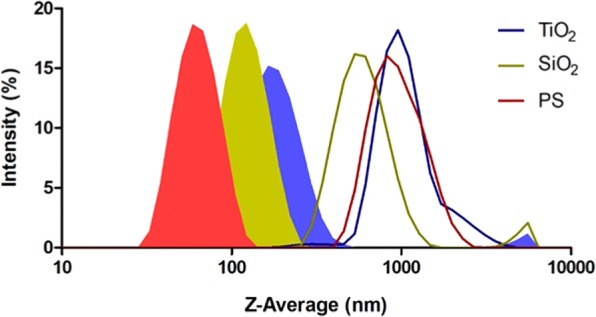
Intensity-weighted size distributions (%) of suspensions containing TiO_2_ (blue), SiO_2_ (ochre) and PS (red) NP at 25 mg/L in Milli-Q water (area filled) and seawater (NSW, 0.45 μm filtered, salinity 35). Logarithmic scale is reported for X-axis, with the minimum value set at 10 nm. Traces are the average of at least 3 independent measurements, edited using Graph Pad Prism 5.

Dynamic Light Scattering (DLS) results from the physico-chemical characterization of NP are summarized in Table 1. NP were well dispersed in Milli-Q water, with Z-Average values below 200 nm and, close to the nominal sizes for SiO_2_ (92 nm *vs*. 109.5 nm) and PS (42 nm *vs*. 55.5 nm), while the formation of small agglomerates was observed for TiO_2_ (25 nm *vs*. 187.2 nm) (Fig. 1). The optimal dispersion and stability in this medium were also confirmed by the polydispersity index (PDI) (in the range of 0.16–0.20) and ζ-potential values (from −30 to −50 mV). In contrast, NP formed larger agglomerates and displayed higher instability in natural seawater (NSW, 0.45 μm filtered) (Table 1, Fig. 1), as shown by higher Z-Average values (in the range of 600–1000 nm), higher PDI values (in the range of 0.27–0.36) and lower absolute ζ-potential values (from −12.2 to −10.2 mV). Nano-scale agglomerates in NSW were observed for SiO_2_, having an average hydrodynamic diameter of 619 ± 23 nm, while micro-scale agglomerates were reached by PS (970 ± 108 nm) and TiO_2_ (1079 ± 108 nm).

**Table 1:** Characterization of TiO_2_, SiO_2_ and PS NPs at 25 mg/L in Milli-Q water and seawater (NSW, 0.45-μm filtered, salinity 35, pH 8.05) used for *A. parkinsoniana* exposure.

	MilliQ			NSW		
	Z-average (nm)	PDI	ζ- potential (mV)	Z-average (nm)	PDI	ζ- potential (mV)
25 nm TiO_2_	187.20 ± 2.58	0.192 ± 0.024	–30.20 ± 2.30	1079 ± 107.70	0.311 ± 0.015	–10.2 ± 1.78
92 nm SiO_2_	109.50 ± 4.98	0.203 ± 0.020	–39.90 ± 1.23	618.9 ± 23.21	0.358 ± 0.055	–12.20 ± 2.86
42 nm PS	55.52 ± 0.82	0.161 ± 0.022	–53.30 ± 1.31	969.80 ± 108.50	0.274 ± 0.031	–10.90 ± 2.75

Z-average (nm), PDI (dimensionless) and ζ-potential (mV) are reported as mean ± SD of 3 independent measurements, performed at a constant temperature of 16 °C.

After a 24-h incubation in NSW and in the presence of foraminifera, DLS measurements of post-treatment suspensions did not allow for a quantitative determination of NP size values, due to the low intensity of scattered light at 1 mg/L concentration. Therefore, the data are reported only as particle size distributions, with an indication of agglomerates presence and normalized to control (Supplementary Fig. S1). Measurements with ESEM (TiO_2_ and SiO_2_) and CLSM (PS) confirmed the presence of nanoscale and microscale agglomerates as shown by DLS (Supplementary Fig. S1).

### Localization of Ti and PS NP in *A. parkinsoniana*

**Figure 2:**
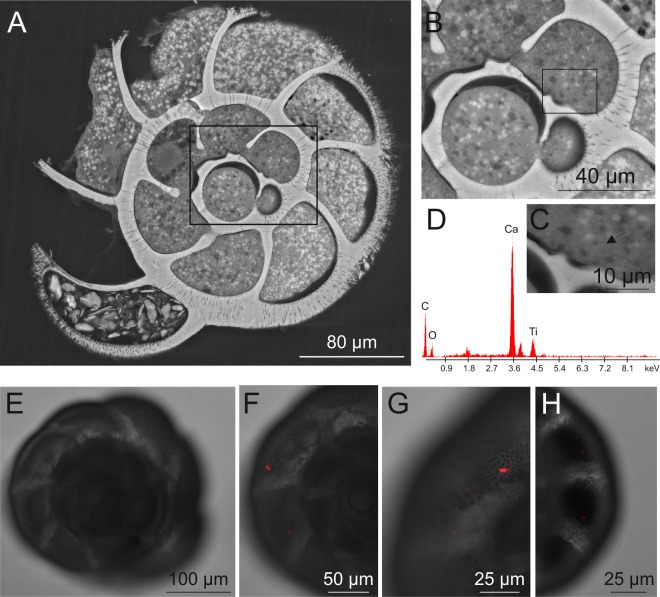
ESEM micrographs (**A–C**) showing the presence of Ti nanoparticles in the treated *A. parkinsoniana* specimens. (**A**) Overview of specimen section where the chamber containing Ti nanoparticles, as dark spots, has been highlighted (black rectangle). (**B**) High magnification of the three last chambers. (**C**) High magnification of the ante-penultimate chamber with the arrow marking the occurrence of Ti nanoparticles. (**D**) EDS spectrum of the agglomerate shown in C. Fluorescent confocal image and bright field overlay micrographs of single optical sections (**E–H**) showing the localization of Flash Red conjugated-PS in *A. parkinsoniana*.

The ESEM-EDS analyses of treated specimens revealed the presence of TiO_2_ NP dark agglomerates in numerous chambers of *A. parkinsoniana*. The distribution of these agglomerates was widespread in the cytoplasmic compartment (Fig. 2A–C). The EDS spectrum of elemental analysis related to these agglomerates revealed the presence of a Ti peak, confirming their chemical nature (Fig. 2D). No TiO_2_ agglomerates were found in control specimens. We also documented with CLSM the cellular uptake and localization of Flash-red conjugated-PS in *A. parkinsoniana* after 24 h at a concentration of 1 mg/L (Fig. 2E–H) that revealed the presence of PS in all chambers from the prolocolus (oldest chamber) to the last (youngest) chamber. No signal of PS-fluorescence was recorded in control specimens.

### Ultrastructural and physiological changes

**Figure 3:**
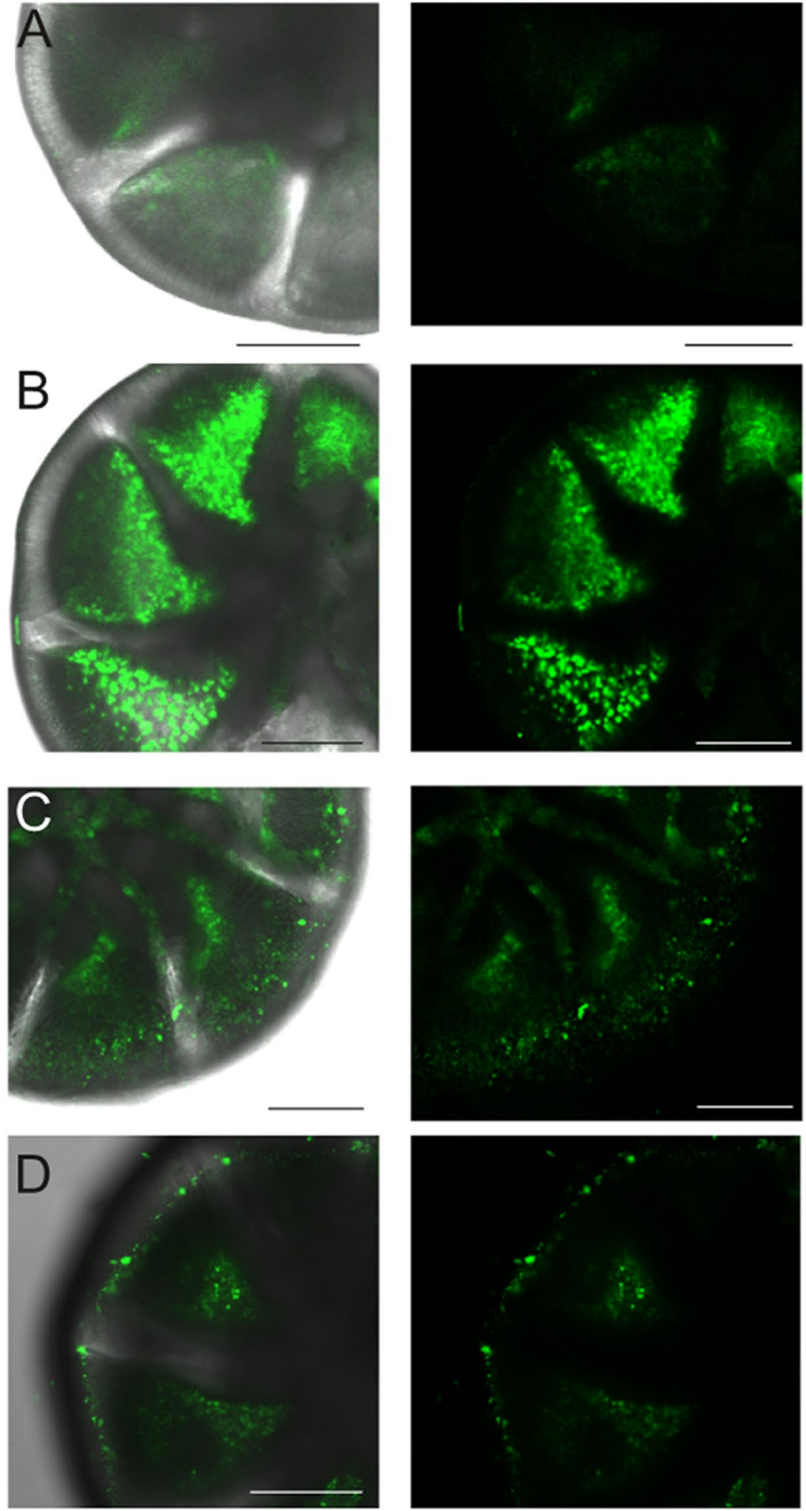
CLSM images showing the effect of NP exposure on ROS production of *A. parkinsoniana* labeled with CellROX®Green. CLSM micrographs of single optical sections showing overlay of green fluorescence-bright field image and fluorescence for control (**A**) and treated TiO_2_ (**B**), SiO_2_ (**C**), PS (**D**) specimens. Scale bar: 50 µm.

The mitochondria of control (untreated) specimens appeared typical, with double membranes and cristae (Supplementary Fig. S2), while the mitochondria of NP-treated specimens were swollen and degraded (Supplementary Fig. S2). The NP treatment induced increased reactive oxygen species (ROS) production in *A. parkinsoniana* (Fig. 3). The free radical formation was localized within any given foraminiferal chamber. Control specimens showed a basal free radical production in intermediate foraminiferal chambers where the fluorescence was dim (Fig. 3A). Foraminiferal specimens incubated with TiO_2_ NP exhibited marked bright fluorescence in well-defined vesicles restricted to the cytoplasm of central chambers (Fig. 3B, Supplementary Figs. S3 and S4). Specimens incubated with SiO_2_ (Fig. 3C) and PS NP (Fig. 3D) exhibited bright fluorescence in all chambers when compared to the dim fluorescence of untreated specimens.

**Figure 4:**
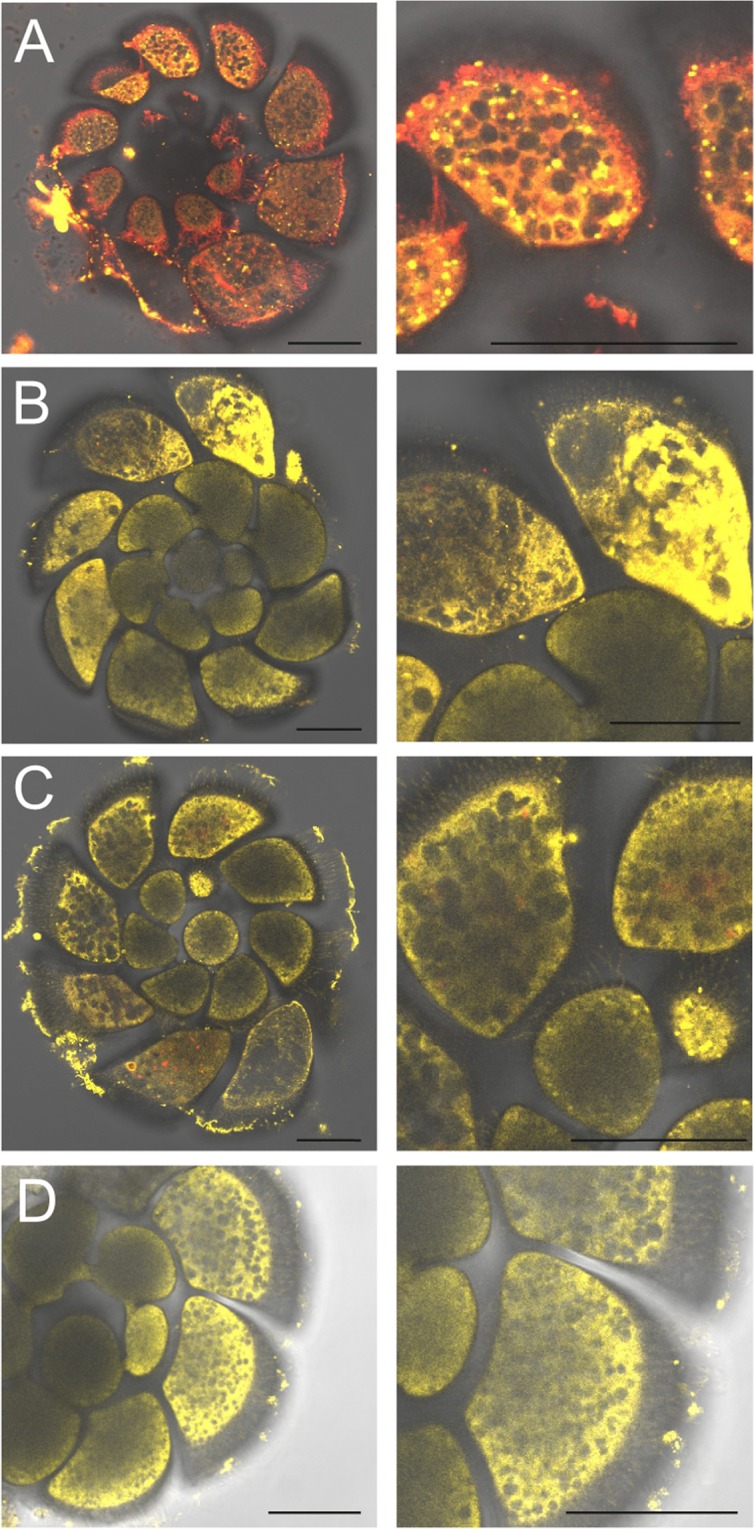
CLSM images showing the effect of NP exposure on lipid distribution of *A. parkinsoniana* labeled with NR. CLSM micrographs of single optical sections showing overlay of NR yellow (neutral lipids) and red (polar lipids) fluorescence for control (**A**) and treated TiO_2_ (**B**), SiO_2_ (**C**), PS (**D**) specimens. Scale bar: 50 µm.

Images obtained with CLSM showed enhanced accumulation of neutral lipids in NP-treated *A. parkinsoniana* compared to control specimens (Fig. 4). In untreated *A. parkinsoniana* specimens, lipids were dominated by the polar form and red color, as evidenced by Nile Red (Fig. 4A), whereas all foraminifera treated with NP exhibited enhanced yellow fluorescence (Fig. 4B–D), suggesting the occurrence of neutral lipids. Neutral and polar lipids occurred as lipid droplets (LD), but different types of NP (e.g. TiO_2_, SiO_2_ and PS) induced an increase of LD as well as an excess of neutral lipids stored in LD of NP-exposed specimens.

## Discussion

Today, the increasing production of nano-based products will inevitably lead to a significant environmental load of NP with consequent impacts on both the natural ecosystem and public health^3,39^. The agglomeration of NP, which results in larger particle size than their primary size, is enhanced in a high ionic-strength medium such as seawater, NP agglomeration is a key phenomenon that is relevant to toxicity in the marine environment^40^. Our results document the formation of large nano-scale agglomerates dispersion in NSW for SiO_2_ (hydrodynamic diameter of 619 ± 23 nm), while micro-scale agglomerates were found for PS (970 ± 108 nm) and TiO_2_ (1079 ± 108 nm). Such different agglomeration behavior in seawater likely results in a different sedimentation rates^24^. Indeed, the heteroagglomeration of NP with NOM and other chemical contaminants promotes settlement to the seafloor and poses a threat to bottom-dwelling and filter-feeding organisms^41^. The increase in the concentration of TiO_2_ NP in sediment is reported to be five orders of magnitude higher than that in surface water^42^. Hence benthos (bottom-dwelling organisms) are at a great toxicological risk and, due their important ecological role, more testing should be prioritized. Eubacteria may be largely protected against the uptake of many types of NP because they do not have mechanisms for transport of colloidal particles across their cell walls^43^. Conversely, eukaryotes (i.e., protists and metazoans) have the ability to internalize nanoscale or microscale particles via endocytosis and phagocytosis^44,45^. Accordingly, carbon nanotubes (CNT) might be ingested by a protozoan and localized within their mitochondria^43^ and, more likely, in phagosomes or lysosomes. The small particle size, the large surface area, and the ability to generate ROS play major roles in NP toxicity^27^. Endocytosis of silver (Ag) NP into food vacuoles and subsequent excretion of their agglomerates has been demonstrated for the protozoan *Tetrahymena thermophila*^46^. Similarly, it was shown that CuO NP were phagocytosed by protozoa^47^ while multicellular aquatic organisms such as daphnids and fish were able to ingest other NP^44^. To date, the majority of aquatic NP studies has been related to their effects on freshwater species; very few studies have been directed towards marine organisms^48^. Even less information exists on the effects of NP on marine benthic unicellular eukaryotes (e.g., benthic foraminifera) which are effectively unstudied.

In the present work, we report, for the first time, the localization and the physiological changes induced by different NP types on the microbial eukaryote *A*. *parkinsoniana*. Based on different qualitative approaches, our results show the compartmentalization of NP within *A. parkinsoniana* and suggest that NP are able to cross the foraminiferal cell membrane. PS NP, in particular, were identified in different foraminiferal chambers by CLSM after a 24-h exposure. A positive fluorescent signal was present in all treated specimens but not in any control *A. parkinsoniana*, demonstrating that the fluorescence was not derived from background biological material (i.e., undigested algae). Our ESEM images coupled with the EDS spectra document the presence of TiO_2_ NP in treated foraminifera as different agglomerates in the cytoplasmic compartment. These data suggest that NP can be ingested and accumulated in benthic organisms, as has already been documented for pelagic species (e.g., fish, shrimp)^14,48^. Given their smaller size and large surface area per unit mass, NP have greater potential of being accumulated, possibly causing deleterious effects in the host^49^. Although this study was not aimed at unravelling the pathway of internalization, the most likely route of NP uptake in benthic foraminifera is active uptake with larger particles such as food and detritus since passive diffusion is highly unlikely considering the NPs agglomerated into micrometer-sized particles. Recently, cytological and ultrastructural modifications of foraminifera resulting from environmental stress have attracted notable interest. These cytological modifications seem to be related to defense mechanisms adopted by foraminifera to protect the cell against potential toxicants^36^. Prior studies have indicated that in at least some benthic foraminifera, potential toxicants like heavy metals promoted cytological modifications, such as lipid vesicle development related to disturbances in the metabolic regulation (see ref. ^35^ for example). Changes to lipid compartments are very important as a parameter for assessing adverse conditions because lipid droplets are hypothesized to sequester contaminants as a means to protect cells^50^. Indeed, a proliferation of lipid droplets, mainly neutral lipids (e.g., esterified cholesterol, triglycerides), has been documented in foraminiferal specimens treated with heavy metals^35,44^. In addition, recent papers have reported the size and concentration-dependent effects of TiO_2_ NP on neutral lipid accumulation^51^. In our experiment, a significant increase in the accumulation of neutral lipids occurred in NP-treated specimens regardless of the NP types, when compared to control specimens. These results are in accordance with findings described for other taxa exposed to similar conditions, such as in *Daphnia similis* as a response to nanowire exposure^52^ and in *Mytilus galloprovincialis* treated with TiO_2_ NP^53^. Interestingly, increasing brightness of Nile Red fluorescence was observed in specimens of *Daphnia galeata* exposed to PS NP, particularly in the brood chamber; this increased brightness was interpreted as a change in lipid storage^54^ or as a significant disturbance of lipid metabolism in the blue mussel^55^. Interestingly, the accumulation of PS NP in lipid storage droplets has been noted in *D. magna*^56^.

The production of ROS is an important component of the physiological response in marine organisms exposed to a variety of factors including pollution^57^. In foraminifera, oxidative stress may induce cellular damage and death as a possible response to different environmental stressors, though they may use oxygen derived from the breakdown of environmentally and metabolically produced H_2_O_2_^58^. In the symbiont-bearing foraminifera *Amphistegina lessonii*, the short-term effects of Zn exposure induced an increase of ROS^59^. Similarly, oxidative stress was reported in *A. gibbosa* after light stress^60^. It has been suggested that NP can enhance the generation of free radicals, ROS, or both, promoting oxidative stress^56^, which is considered to be a key factor involved in nanotoxicity. Generally, metal NP stimulate the toxicity mediated by free radicals through Fenton-type reactions, while CNT induces ROS generation mediated by mitochondrial damage^61^. Because ROS production is an important event during NP-induced damage, the process must be well characterized to predict NP-induced toxicity^62^. Our results show that all three types of NP tested induced free radical production. More specifically, the most ROS production is shown in specimens treated with TiO_2_, where the signal is particularly bright, and to a lesser extent in SiO_2_ labeled specimens. Previous *in vivo* studies have demonstrated the capability of TiO_2_ NP to induce oxidative stress in different organisms (e.g., mussels, abalone, humans) by increasing the production of lipid peroxidation and nitric oxide (see ref. ^48^ for example).

Our study reveals, for the first time, the effects on a benthic unicellular eukaryote living in marine sediment to three kinds of ENM. It suggests that ENM have deleterious effects on benthic organisms, which are commonly neglected in the research despite representing an important link in the trophic web. The ability to internalize ENM coupled with ENM persistence may also promote the transfer and biomagnification of NP through the food web. In light of the increasing occurrence of ENM in marine environments, the evaluation of their cytotoxicity and the internalization capability of bottom-dwelling organisms is an essential step to predict future exposure scenarios. These benthic marine microbial eukaryotes may represent a new model by which to assess future NP impacts scenarios in the neglected marine benthos.

## Methods

### Sampling of sediment and specimens

Sediment and natural seawater (NSW) samples were collected at a coastal site (43°33′54″ N, 13°39′52″ E) off the Monte Conero area (Adriatic Sea, Italy) in front of the terrestrial Regional Natural Park of Conero. In the laboratory, the sediment was sieved over 125 µm and 250 μm screens with NSW, and the residue obtained was used for collecting living benthic foraminiferal specimens of *Ammonia parkinsoniana*. The presence of reticulopodial activity has been used as viability criterion. The physico-chemical characteristics of the NSW were a salinity of 35, a pH of 8.05, a dissolved oxygen concentration of 6.8 mg/L, and a chlorophyll-*a* content of 0.28 µg/L.

### Physico-chemical characterization of nanoparticles (NP)

Nanoscale TiO_2_ (nominal diameter of 25 nm) namely Aeroxide© with a declared purity of 99.9% was supplied by Eigenmann & Veronelli (Milan, Italy), SiO_2_ (100 nm) was provided by the Centre for BioNano Interactions, University College of Dublin (Dublin, Ireland), and Flash Red conjugated-PS (42 nm, ex/em 660/690) was purchased from Bangs Laboratories Inc. (Fishers, IN, USA) (Lot number 12210). TiO_2_ stock suspension (10 mg/mL) was prepared from Aeroxide© white powder, while SiO_2_ and PS stocks were both supplied in deionized water containing 10 mg/mL.

Characterization of TiO_2_, SiO_2_ and PS NP at 25 mg/L was performed through Dynamic Light Scattering (DLS) using a Zetasizer Nano ZS90 (Malvern Instruments, UK), equipped with a 633 nm laser and a 90° backscattering angle, combined with Zetasizer Nano Series Software Version 7.02 (Particular Sciences, UK). For each NP, the material refractive index and absorption were set following the guidelines provided by Malvern Instruments. Z-average (nm), polydispersity index (PDI, dimensionless) and Zeta (ζ-) potential (mV), referred to average hydrodynamic diameter, size distribution and electro-kinetic potential (i.e. surface charge) respectively, and were measured soon after the dispersion (at 0 h). The ζ-potential, measured by electrophoretic mobility, is considered an indirect way to measure NP tendency to agglomerate in the medium. Three measurements, each containing 11 runs of 10 seconds for Z-average and PDI and 20 runs for ζ-potential, were carried out at a constant temperature of 16 °C.

For this purpose, NP working suspensions (at 25 mg/L) in 0.45 μm filtered NSW were freshly prepared from stock solutions and quickly vortexed prior to use, but not sonicated. NP suspensions in Milli-Q water were also analyzed as a reference for optimal dispersion. NSW with no added NPs was analyzed as a control.

As the size of NPs can be biased by SEM analyses that cause shrinkage, charge acquisition and alteration, an ESEM, which operates at a low-pressure gaseous environment of 10–50 Torr with high humidity and without coating, was used to image NPs in their natural state without modification. NPs were dispersed in NSW at a concentration of 1 mg/L for 24 h, after which a 1 mL droplet of the suspensions for each NP was deposited by a precision pipette on the sample holder. The chamber was then immediately closed and pumped down to 900 Pa. This value is far above the dew point of water (100% humidity corresponds to 709 Pa at this temperature). The microscope optics were then aligned and optimized, and the chamber pressure was slowly reduced in intervals of 20 Pa with resting periods of 5 min for every 100 Pa, causing gradual evaporation of the water.

### Exposure design and experimental set-up

Stock suspensions of TiO_2_ and SiO_2_ in deionized water were sonicated with a 50% on/off cycle for 45 minutes at 100 W using a UP200S Hielscher Ultrasonic device (Teltow, Germany), and the resulting dispersion was being cooled in an ice bath and immediately diluted in NSW at 1 mg/L. PS suspensions (1 mg/L) were prepared in NSW from stock suspensions that were quickly vortexed prior to use but not sonicated.

Specimens were collected from culture batches that were normally kept at 15 °C and gradually raised to 18 °C in the week preceding the experiment. These concentrations were set to be low enough to not induce lethal effects due to exposure, and to allow the detection of physiological changes and the presence of NPs. Specimens of *A. parkinsoniana* were randomly picked from the pool to be exposed to 1 mg/L of NP suspensions (see above) for 24 h; control samples were run in parallel, incubated for the same duration in NSW. A total of 360 living individuals of *A. parkinsoniana* were transferred to six-well tissue culture plates (UltraCruz®) and covered with 5 mL of NP suspension. At least twenty individuals were placed in each well. Plates were then left in a controlled environment, at 18 °C under a 12:12 h light-dark cycle. The exposure time was kept short in order to avoid feeding foraminiferal specimens and to prevent the uptake of NPs absorbed by algae, and to limit autofluorescence in the case of NP PS. All experimental conditions were replicated three times by using 5 specimens for each fluorogenic probe in the CLSM analysis and 10 specimens for TEM and ESEM-EDS analyses.

### Transmission electron microscopy (TEM) and environmental scanning electron microscopy coupled with energy dispersive X-ray spectrometry (ESEM-EDS): specimen preparation and analysis

*Ammonia parkinsoniana* preparation for TEM and ESEM analyses is described in^44^. Briefly, samples were fixed with 2.5% glutaraldehyde (TAAB, England, UK) in Artificial Sea Water (ASW) for 3 h at 4 °C. Specimens for TEM analysis were decalcified with 0.1 M EDTA for 36 h. After 5 washings with ASW, all specimens, both those for TEM and those for ESEM analysis, were post-fixed with 1% osmium tetroxide (OsO_4_; EMS, Hatfield, PA) in ASW for 2 h at room temperature. Specimens were then dehydrated in a graded series of ethanol baths, from 50 to 100%, immersed twice in propylene oxide (10 min each; EMS, Hatfield, PA), and embedded in epoxy resin (Durcupan Araldite, SIGMA, UK). Foraminifera were ultimately sectioned using an ultramicrotome (LKB, 2088 Ultratome^®^V). Thick sections of 1 μm were stained with 1% toluidine blue in distilled water at 60 °C to provide an overview at the light-microscope level. For the TEM analysis, thin sections (100 nm), collected on 300-mesh nickel grids, were stained with 3% aqueous uranyl acetate and Reynold’s lead citrate solutions and finally observed with a Philips CM10 electron microscope at 80 kV.

For the ESEM analysis, embedded specimens used for TEM were observed, as a whole, with an environmental scanning electron microscope (FEI ESEM, Quanta 200) to qualitatively characterize the presence of Ti. The localization of Si was not performed as it is a natural component of the sediment and it could not have disentangled the NP SiO_2_. The ESEM, coupled with energy dispersive X-ray spectrometry (EDS), was used to assess the elemental composition of particles in the cytoplasm. Observations were conducted in low vacuum (0.2–1.2 Torr) at 10-mm working distance using secondary and backscattered electron modes with energy varying from 12 to 25 kV. A live counting time of 100 seconds, with spots’ mode from 3 to 5, was used for elemental mapping.

### Confocal laser scanning microscopy (CLSM) analyses

CLSM is a non-destructive method that allows the evaluation of physiological changes and the occurrence of fluorescent NPs. Selected specimens of *A. parkinsoniana* (treated samples and control samples) were incubated with Nile Red (NR) or CellROX^®^Green and analyzed with CLSM. To check and account for autofluorescence and for the localization of Flash Red conjugated-PS, unstained samples were examined. The probes were obtained from Thermo Fisher Scientific (Massachusetts, USA). A Leica Microsystems TCS SP5 II CLSM with 488, 543 and 663 nm excitation illumination and oil-immersion objectives was used. The images were further processed, as required, in ImageJ software (National Institutes of Health, Bethesda, MD, USA).

For NR microscopy, *A. parkinsoniana* specimens were fixed in 2% paraformaldehyde for 2 hours, then washed in ASW and decalcified with EDTA (0.1 M) for 48 h to remove the foraminiferal test. Following decalcification, the specimens were rinsed in ASW and transferred to MatTek glass bottom chambers (MatTek Corporation, Ashland, MA), and NR was added at the final concentration of 3 μg/ml for 40 min at RT. Using CLSM, specimens were subject to blue excitation (488 nm) and analyzed separately for yellow and red emissions.

CellROX^®^Green is a membrane-permeable probe used to detect reactive oxygen species (ROS) within a cell. This fluorogenic probe is almost non-fluorescent when in a reduced state and shows a bright fluorescent signal when oxidized. Thus, untreated specimens with a low production of ROS show a low fluorescence signal, whereas when the production of ROS increases due to stress conditions, cells show a higher fluorescence. For CellROX^®^Green microscopy, living *A. parkinsoniana* specimens were directly incubated in 5 µM CellROX^®^Green for 60 min at RT and subject to blue excitation (500 nm) and analyzed for green fluorescent emission (525 nm) using CLSM^63^.

## Supplementary information

Supplementary figures

## Data Availability

All data can be obtained from the corresponding author upon request.
