# Supplementary material for: Nanoparticle-Biological Interactions in a Marine Benthic Foraminifer
Source: Sci Rep. 2019 Dec 19;9:19441. doi: 10.1038/s41598-019-56037-2 (PMC6923483; doi:10.1038/s41598-019-56037-2)
Supplement: Supplementary file 1 — Supplementary figures [file 41598_2019_56037_MOESM1_ESM.docx]

**Nanoparticle-Biological Interactions in a Marine Benthic Foraminifer**

Caterina Ciacci^1*^, Margot V. Grimmelpont^2,3^, Ilaria Corsi^4^, Elisa Bergami^4^, Davide Curzi^1^, Debora Burini^1^, Vincent M.P. Bouchet^2^, Patrizia Ambrogini^1^, Pietro Gobbi^1^, Yurika Ujiié^5^, Yoshiyuki Ishitani^6^, Rodolfo Coccioni^3^, Joan M. Bernhard^7^, Fabrizio Frontalini^3^

^1^Università degli Studi di Urbino, Department of Biomolecular Science, Urbino (Italy)

^2^Univ. Lille, CNRS, Univ. Littoral Cote d’Opale, UMR 8187, LOG, Laboratoire d'Océanologie et de Géosciences, Wimereux (France)

^3^Università degli Studi di Urbino, Department of Pure and Applied Science, Urbino (Italy)

^4^Università degli Studi di Siena, Department of Physical, Earth and Environmental Sciences, Siena (Italy)

^5^Kochi University, Center for Advanced Marine Core Research, Nankoku (Japan)

^6^ University of Tsukuba, Section Center for Computational Sciences, Tsukuba (Japan)

^7^Geology and Geophysics Department, Woods Hole Oceanographic Institution, Woods Hole, MA (USA)

*caterina.ciacci@uniurb.it


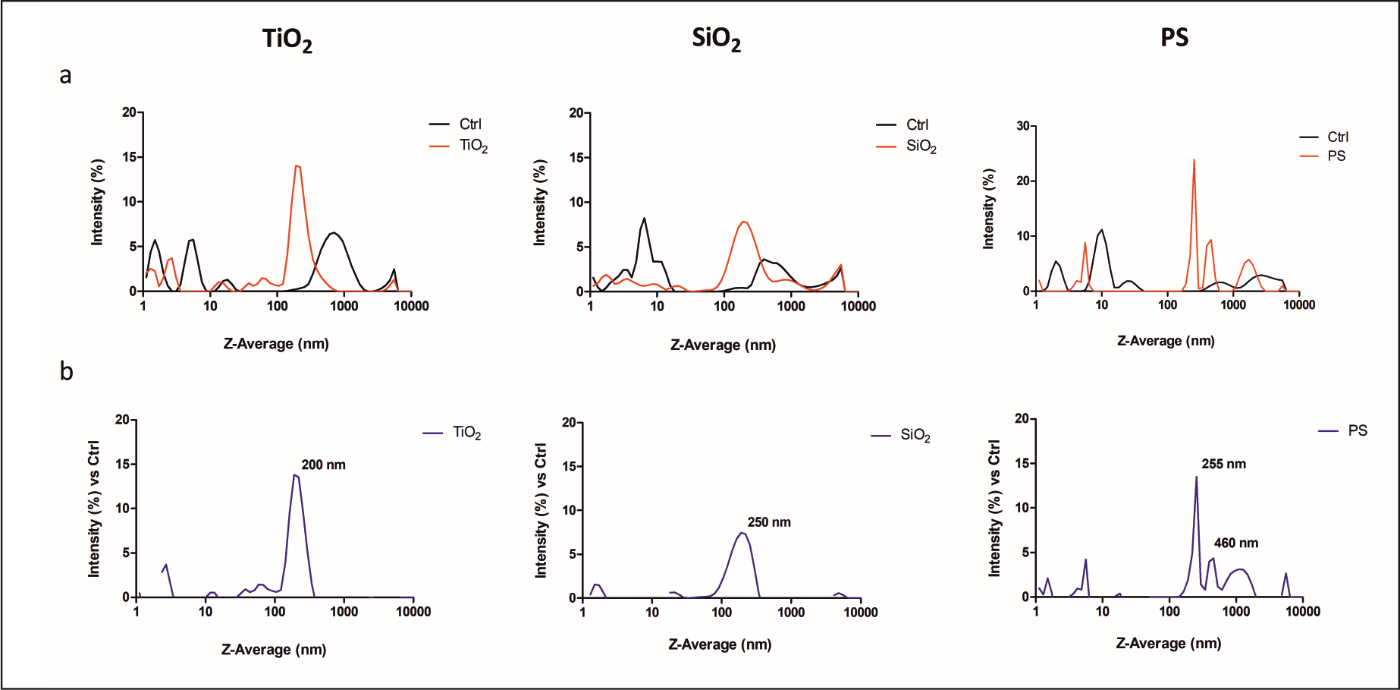


**Supplementary Figure S1.** Characterization of post-treatment suspensions by DLS. Intensity-weighted size distributions (%) of suspensions in NSW (0.45-μm filtered, salinity 35, pH 8.05) from the control group (seawater only) and containing TiO_2_, SiO_2_ and PS NPs at 1 mg/L collected after 24 h of exposure of *A. parkinsoniana* (A) and normalised to control (B). Note logarithmic scale of X-axis. The graphs are the average of at least 3 independent measurements, edited using Graph Pad Prism 5.


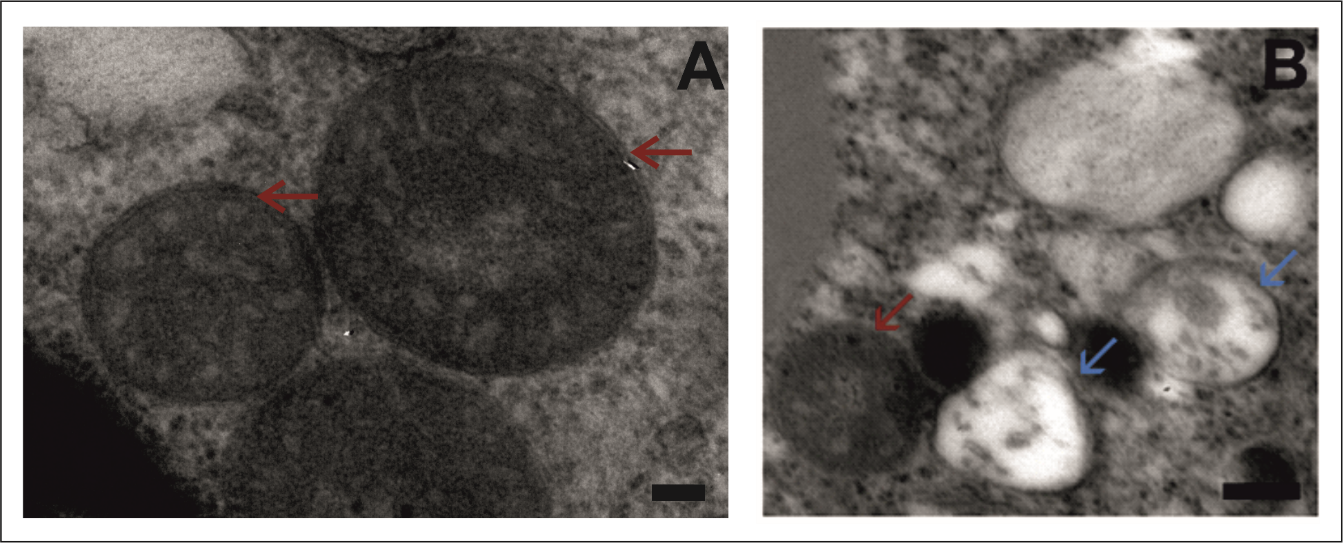


**Supplementary Figure S2.** TEM micrographs of *Ammonia parkinsoniana*. High magnification views of foraminiferal cytoplasm of control (A) and Ti-treated (B) specimens, where intact (red arrow) and degraded (blue arrow) mitochondria were observed, respectively. Scale bars: A-B: 100 nm.


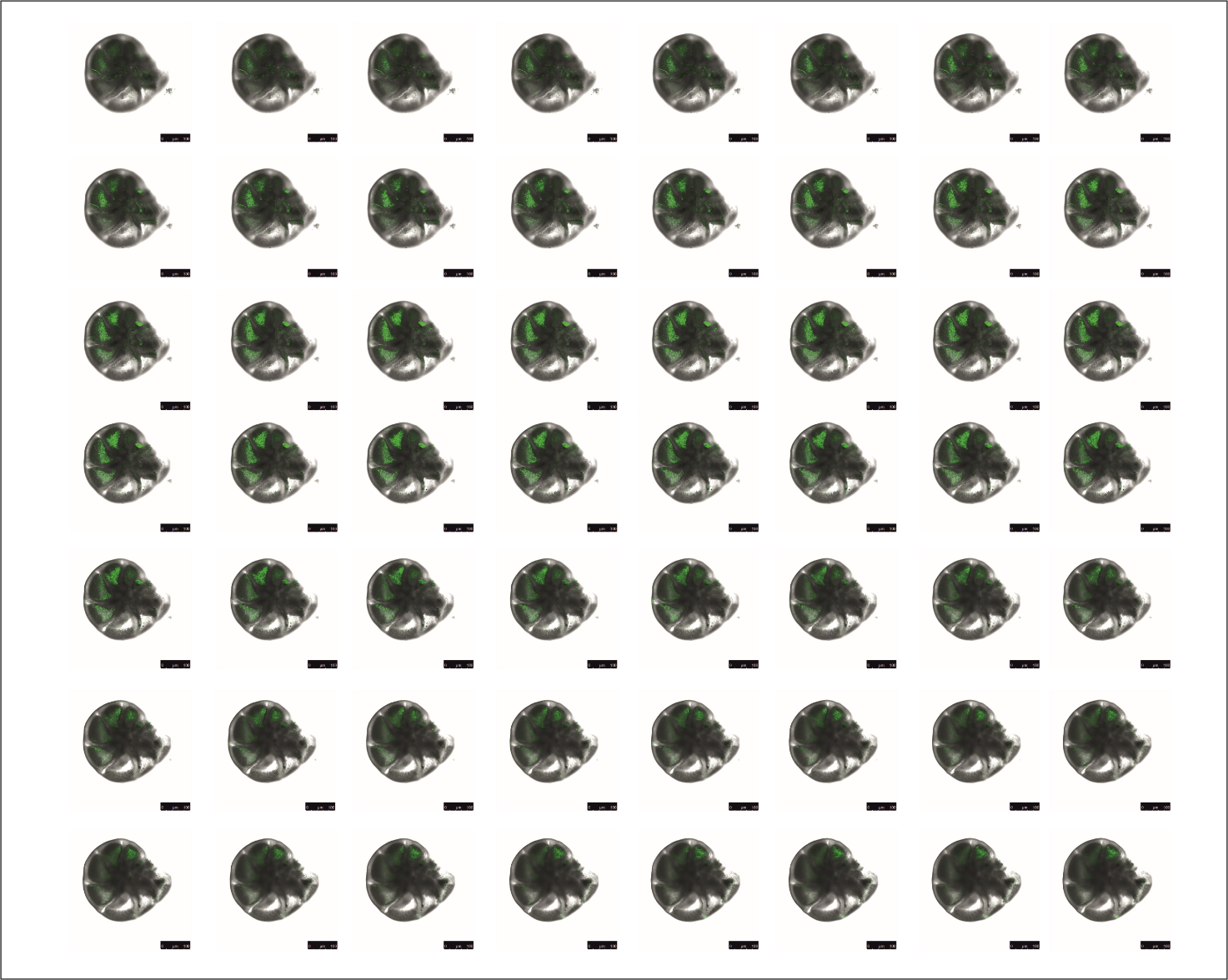


**Supplementary Figure S3.** CLSM images on the effect of TiO_2_ exposure on ROS production of *A. parkinsoniana* labelled with CellROX®Green. CLSM micrographs of sequential single optical sections taken at 0.69 µm interval showing overlay of green fluorescence-bright field image. Scale bar: 100 µm.


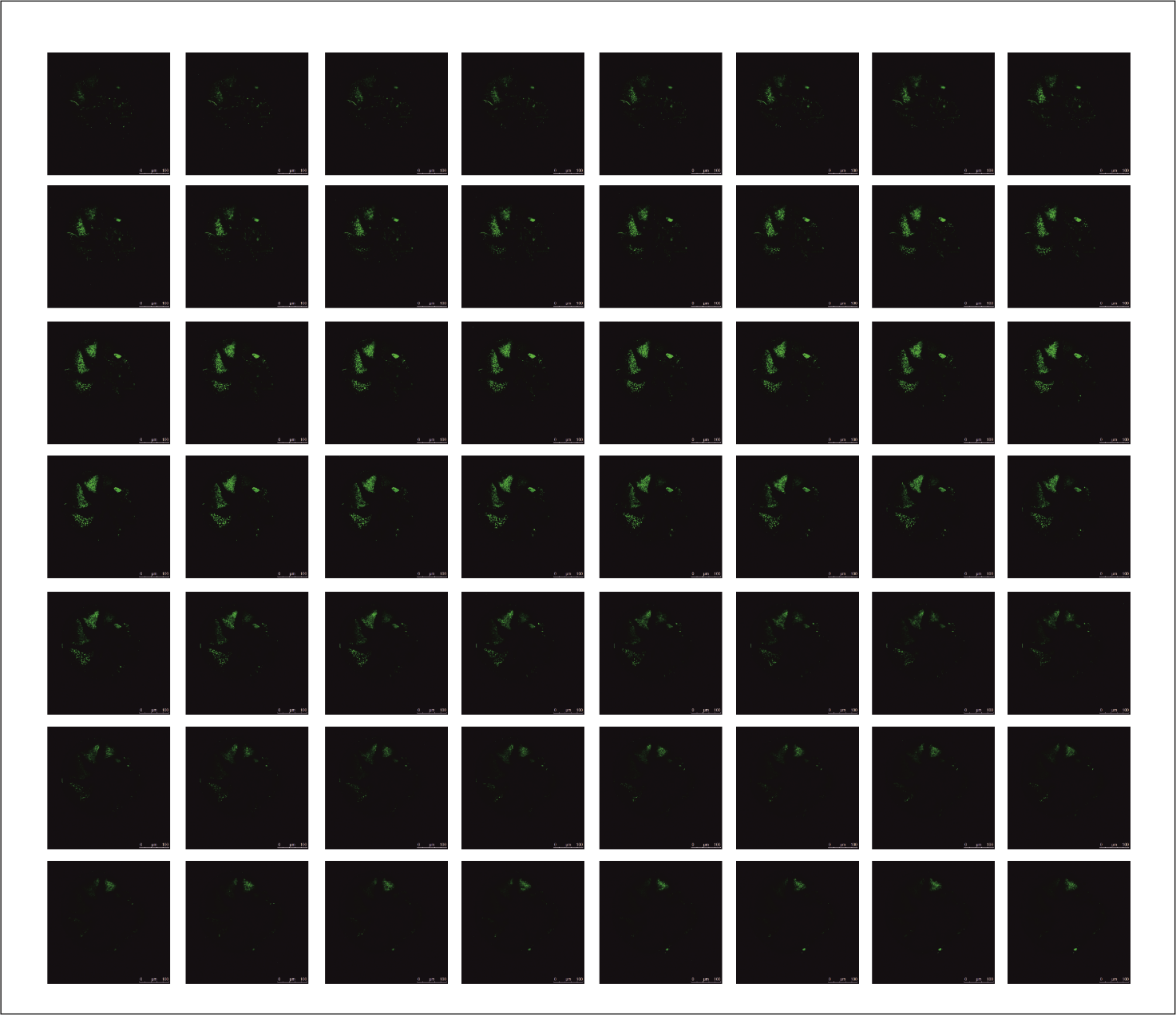


**Supplementary Figure S4.** CLSM images on the effect of TiO_2_ exposure on ROS production of *A. parkinsoniana* labelled with CellROX®Green. CLSM micrographs of sequential single optical sections taken at 0.69 µm interval showing only green fluorescence. Scale bar: 100 µm.
